# Author Correction: Targeted apoptosis of macrophages and osteoclasts in arthritic joints is effective against advanced inflammatory arthritis

**DOI:** 10.1038/s41467-026-75447-1

**Published:** 2026-07-09

**Authors:** Caifeng Deng, Quan Zhang, Penghui He, Bin Zhou, Ke He, Xun Sun, Guanghua Lei, Tao Gong, Zhirong Zhang

**Affiliations:** 1 Key Laboratory of Drug-Targeting and Drug Delivery System of the Education Ministry, Sichuan Engineering Laboratory for Plant-Sourced Drug and Sichuan Research Center for Drug Precision Industrial Technology, West China School of Pharmacy, Sichuan University, Chengdu, 610064 China; 2 Department of Orthopaedics, Xiangya Hospital, Central South University, Changsha, 410008 China; 3 Institute of Materia Medica, School of Pharmacy, Chengdu Medical College, Chengdu, 610500 China; 4 Development and Regeneration Key Lab of Sichuan Province, Department of Pathology, Department of Anatomy and Histology and Embryology, Chengdu Medical College, Chengdu, 610500 China; 5 Hunan Key Laboratory of Joint Degeneration and Injury, Changsha, 410008 China; 6 National Clinical Research Center of Geriatric Disorders, Xiangya Hospital, Central South University, Changsha, 410008 China

Correction to: *Nature Communications* 10.1038/s41467-021-22454-z, published online 12 April 2021

In the version of this article initially published, in Fig. 8d, H&E row, Normal and Anti-TNF columns, incorrect representative images were shown, while in Supplementary Fig. 1, Toluidine row, Normal column, an incorrect representative image was shown. The figures have been replaced in the HTML and PDF versions of the article. For comparison, the original figures are available as Supplementary Information accompanying this article.

## Supplementary information

Original Fig. 8, Supplementary Fig. 1

