# Supplementary material for: Targeted apoptosis of macrophages and osteoclasts in arthritic joints is effective against advanced inflammatory arthritis
Source: Nat Commun. 2021 Apr 12;12:2174. doi: 10.1038/s41467-021-22454-z (PMC8042091; doi:10.1038/s41467-021-22454-z)
Supplement: Supplementary file 1 — Supplementary Information [file 41467_2021_22454_MOESM1_ESM.pdf]

Supplementary Information

Targeted apoptosis of macrophages and osteoclasts in arthritic joints is effective against advanced inflammatory arthritis

Caifeng Deng[1,2], Quan Zhang[3,4], Penghui He[1], Bin Zhou[2,5], Ke He[2,5], Xun Sun[1], Guanghua Lei[2,5,6,*], Tao Gong[1,*] and Zhirong Zhang[1]

[1] Key Laboratory of Drug-Targeting and Drug Delivery System of the Education Ministry, Sichuan Engineering Laboratory for Plant-Sourced Drug and Sichuan Research Center for Drug Precision Industrial Technology, West China School of Pharmacy, Sichuan University, Chengdu 610064, China.

[2] Department of Orthopaedics, Xiangya Hospital, Central South University, Changsha 410008, China.

[3] Institute of Materia Medica, School of Pharmacy, Chengdu Medical College, Chengdu 610500, China.

[4] Development and Regeneration Key Lab of Sichuan Province, Department of Pathology, Department of Anatomy and Histology and Embryology, Chengdu Medical College, Chengdu 610500, China.

[5] Hunan Key Laboratory of Joint Degeneration and Injury, Changsha 410008, China.

[6] National Clinical Research Center of Geriatric Disorders, Xiangya Hospital, Central South University, Changsha 410008, China.

[*] **Corresponding author**

Prof. Guanghua Lei

87 Xiangya Road, Changsha, Hunan, China, 410008

Email: lei_guanghua@csu.edu.cn

Prof. Tao Gong

No.17, Block 3, Southern Renmin Road, Chengdu, China, 610041

Email: gongtaoy@126.com

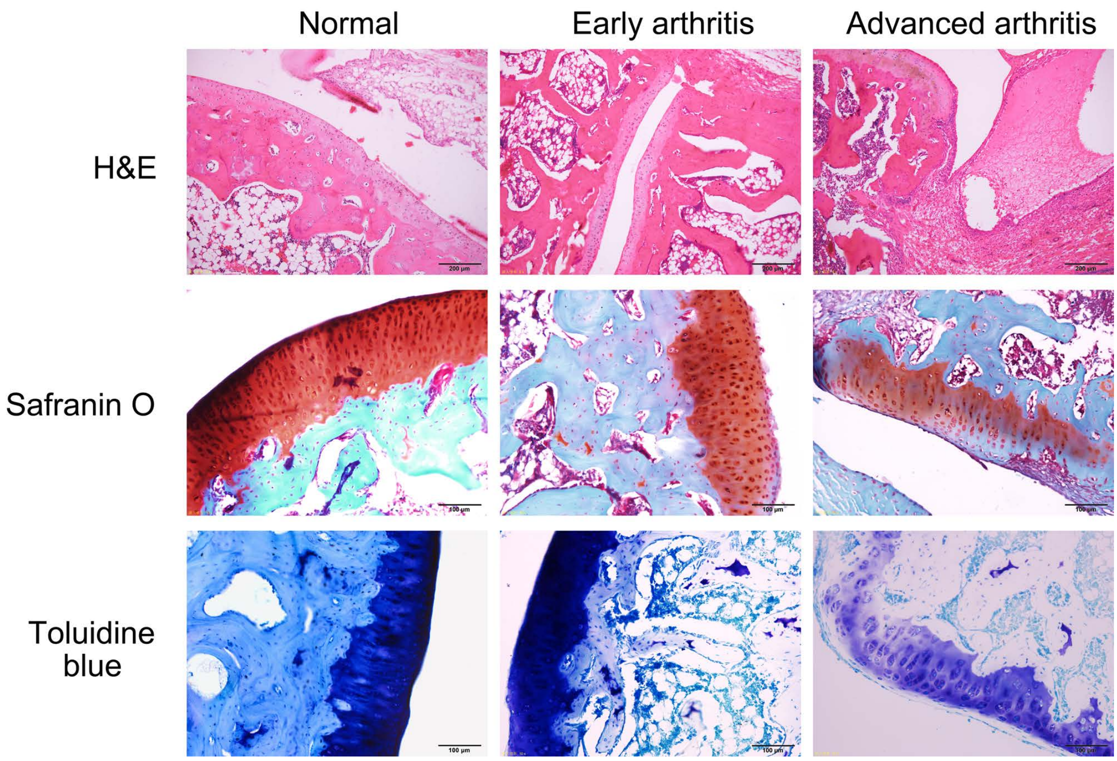

Supplementary Figure 1: Histopathology evaluation of rat ankle joints. The ankle joints from normal rats, AIA rats with early-stage arthritis and AIA rats with late-stage arthritis were stained by H&E (Scale bar = 200 μm), Safranin-O (Scale bar = 100 μm) and toluidine blue (Scale bar = 100 μm) (*n* = 5 independent animals). H&E, hematoxylin-eosin.

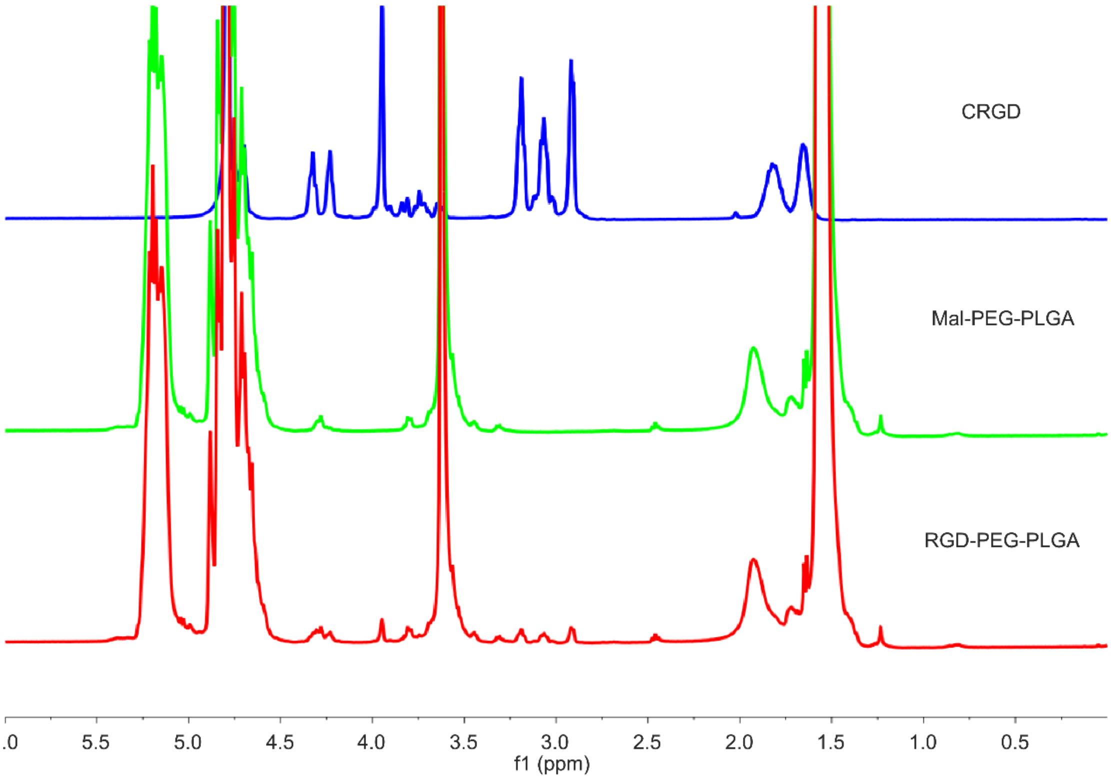

Supplementary Figure 2: ^{1}H-NMR spectra of CRGD, Mal-PEG-PLGA and synthesized RGD-PEG-PLGA. CRGD, cysteine-arginine-glycine-aspartic acid tetrapeptide sequence; Mal-PEG-PLGA, maleimide-poly (ethylene glycol)-poly (D, L-lactide-co-glycolide 50/50) copolymer; RGD-PEG-PLGA, RGD peptide-modified PEG-PLGA.

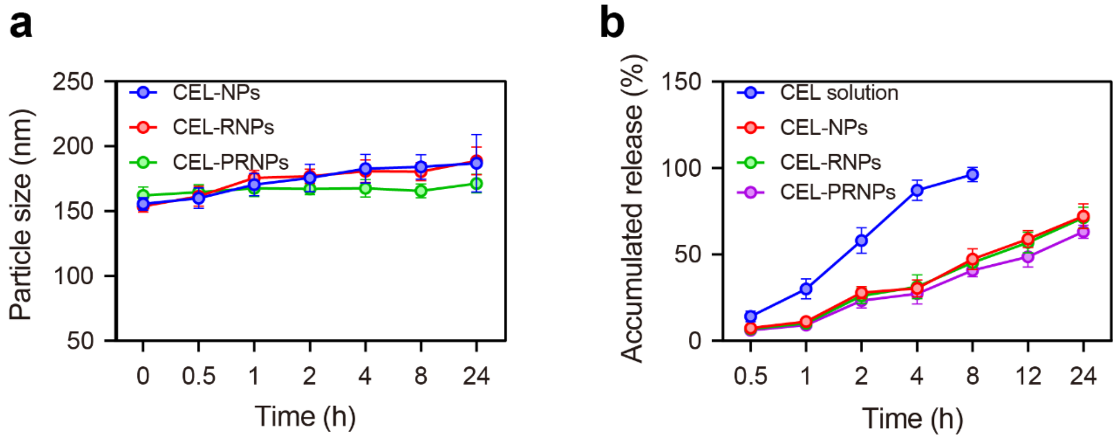

Supplementary Figure 3: Serum stability and *in vitro* drug release behavior of CEL-loaded nanoparticles. (a) The serum stability of CEL-NPs, CEL-RNPs and CEL-PRNPs during 24-h incubation with 10% FBS at 37 °C. Data represent mean ± SD (*n* = 3 independent samples). (b) Cumulative CEL release from CEL-NPs, CEL-RNPs and CEL-PRNPs in PBS at 37 °C. Data represent mean ± SD (*n* = 3 independent samples). CEL, celastrol; CEL-NPs, CEL-loaded poly (D,

L-lactide-co-glycolide) (PLGA) nanoparticles; CEL-RNPs, CEL-loaded RGD peptide-modified PLGA nanoparticles; CEL-PRNPs, CEL-loaded matrix metalloproteinase 9 (MMP9)-cleavable polyethylene glycol (PEG)- and RGD peptide-modified PLGA nanoparticles.

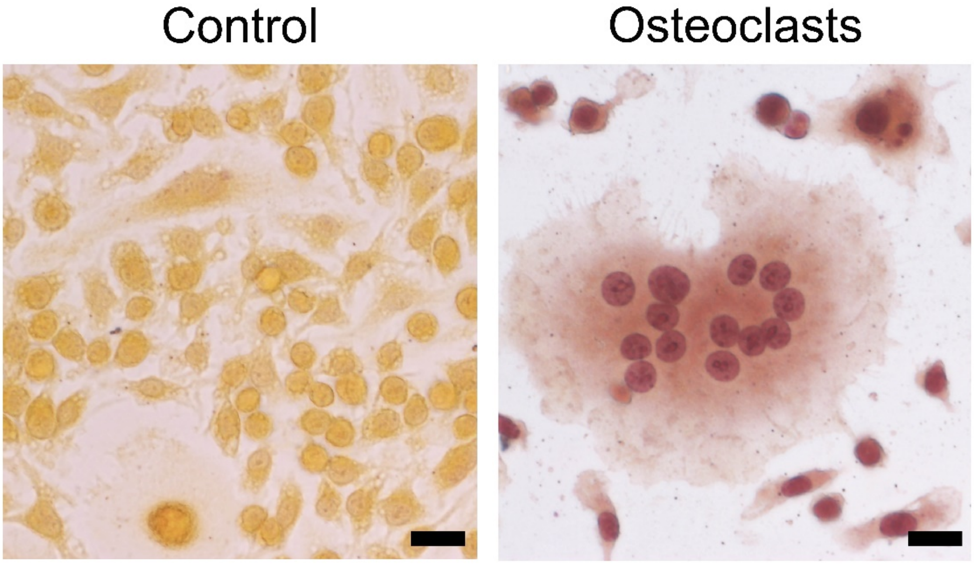

Supplementary Figure 4: Tartrate-resistant acid phosphatase (TRAP) assay. BMMs treated with 30 ng/mL M-CSF for 4 days were used as control. BMMs treated with 100 ng /mL of RANKL and 30 ng/mL of M-CSF for 4 days to induce osteoclast (Scale bar = 50 µm) (*n* = 3 independent samples).

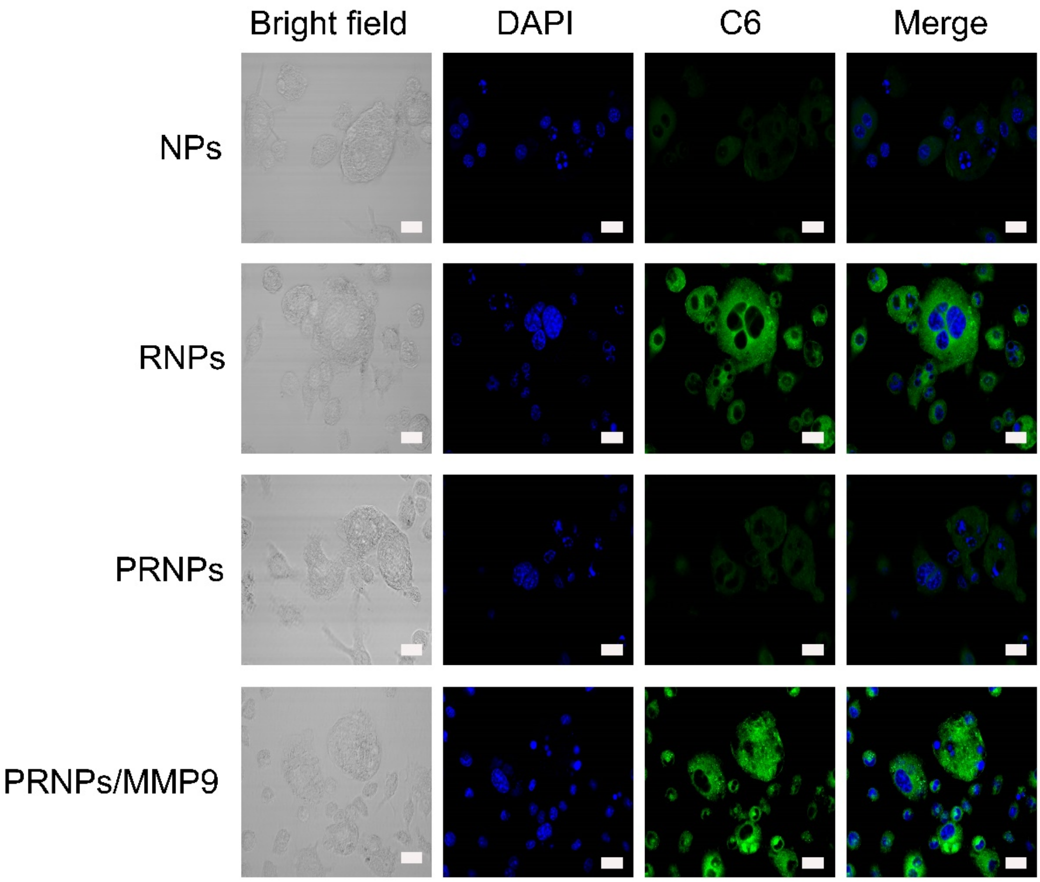

Supplementary Figure 5: Confocal images of cellular uptake on OCs. Confocal images (Scale bar = 50 µm) showing the cellular uptake of various coumarin 6 (C6) loaded nanoparticles on BMMs derived OCs (BMMs with M-CSF and RANKL) (*n* = 3 independent samples). C6, coumarin 6; DAPI, 2-(4-Amidinophenyl)-6-indolecarbamidine dihydrochloride; MMP9, matrix metalloproteinase 9; NPs, poly (D, L-lactide-co-glycolide) (PLGA) nanoparticles; RNPs, RGD peptide-modified PLGA nanoparticles; PRNPs, matrix metalloproteinase 9 (MMP9)-cleavable polyethylene glycol (PEG)- and RGD peptide-modified PLGA nanoparticles.

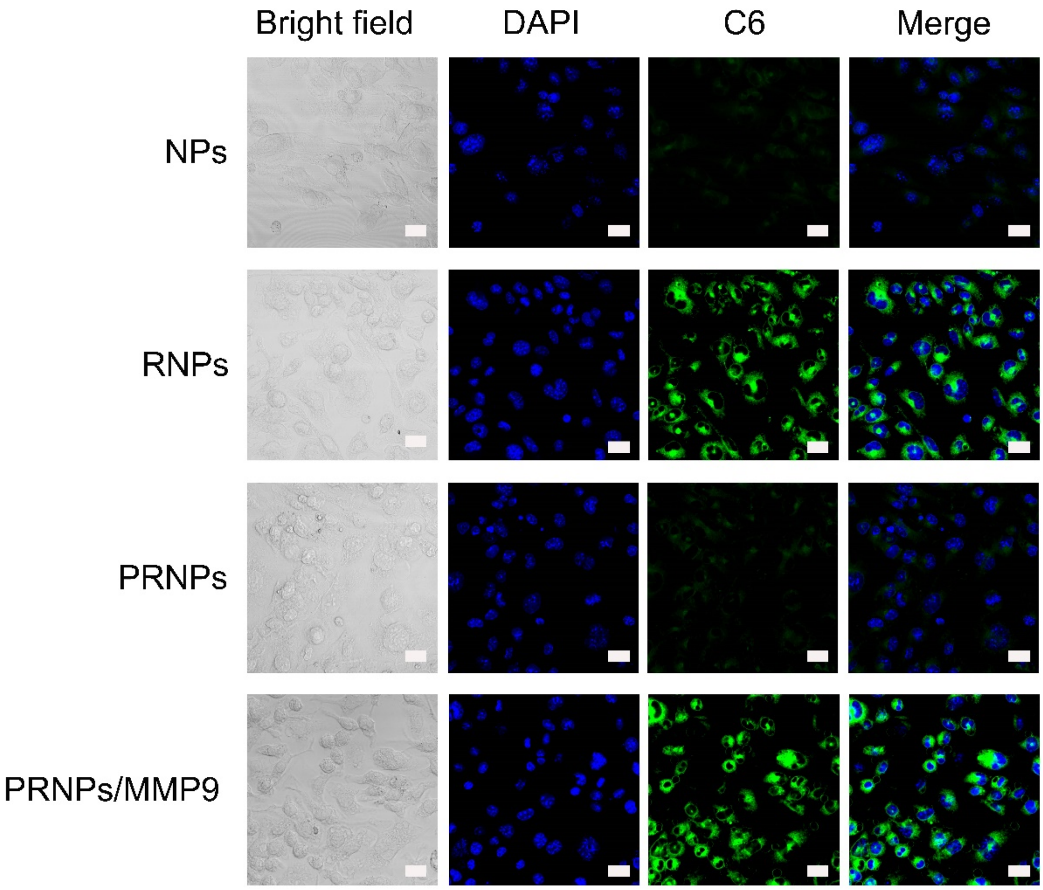

Supplementary Figure 6: Confocal images of cellular uptake on LPS-activated macrophages. Confocal images (Scale bar = 50 μm) showing the cellular uptake of C6-NPs, C6-RNPs or C6-PRNPs on activated macrophages (BMMs with LPS) (n = 3 independent samples). C6, coumarin 6; DAPI, 2-(4-Amidinophenyl)-6-indolecarbamidine dihydrochloride; MMP9, matrix metalloproteinase 9; NPs, poly (D, L-lactide-co-glycolide) (PLGA) nanoparticles; RNPs, RGD peptide-modified PLGA nanoparticles; PRNPs, MMP9-cleavable polyethylene glycol (PEG)- and RGD peptide-modified PLGA nanoparticles.

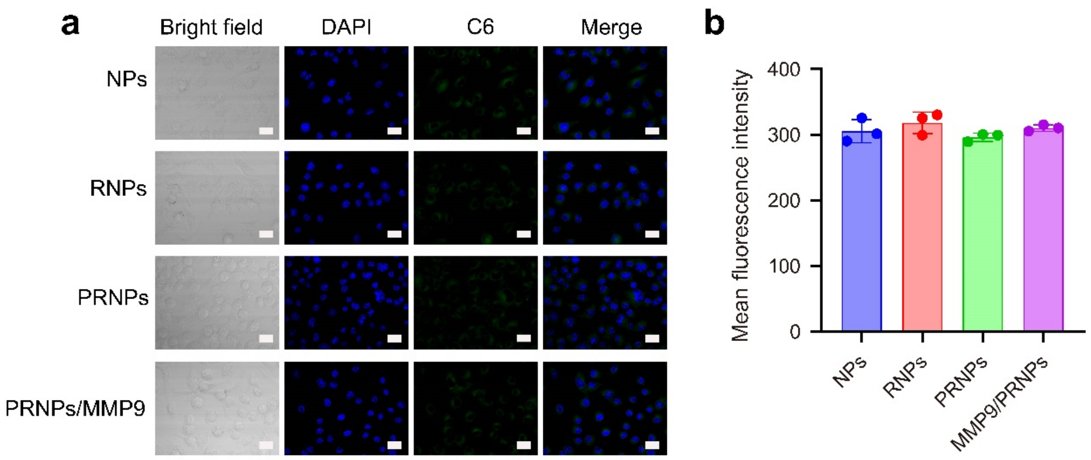

Supplementary Figure 7: Confocal images of cellular uptake on BMMs. (a) Confocal images (Scale bar = 50 µm) showing the cellular uptake of C6-loaded NPs, RNPs or PRNPs on BMMs (*n* = 3 independent samples). (b) Quantitative analysis of the cellular uptake of C6-loaded NPs, C6-loaded RNPs or C6 loaded PRNPs on BMMs. Data represent mean ± SD (*n* = 3 independent samples). C6, coumarin 6; DAPI, 2-(4-Amidinophenyl)-6-indolecarbamidine dihydrochloride; MMP9, matrix metalloproteinase 9; NPs, poly (D, L-lactide-co-glycolide) (PLGA) nanoparticles; RNPs, RGD peptide-modified PLGA nanoparticles; PRNPs, MMP9-cleavable polyethylene glycol (PEG)- and RGD peptide-modified PLGA nanoparticles.

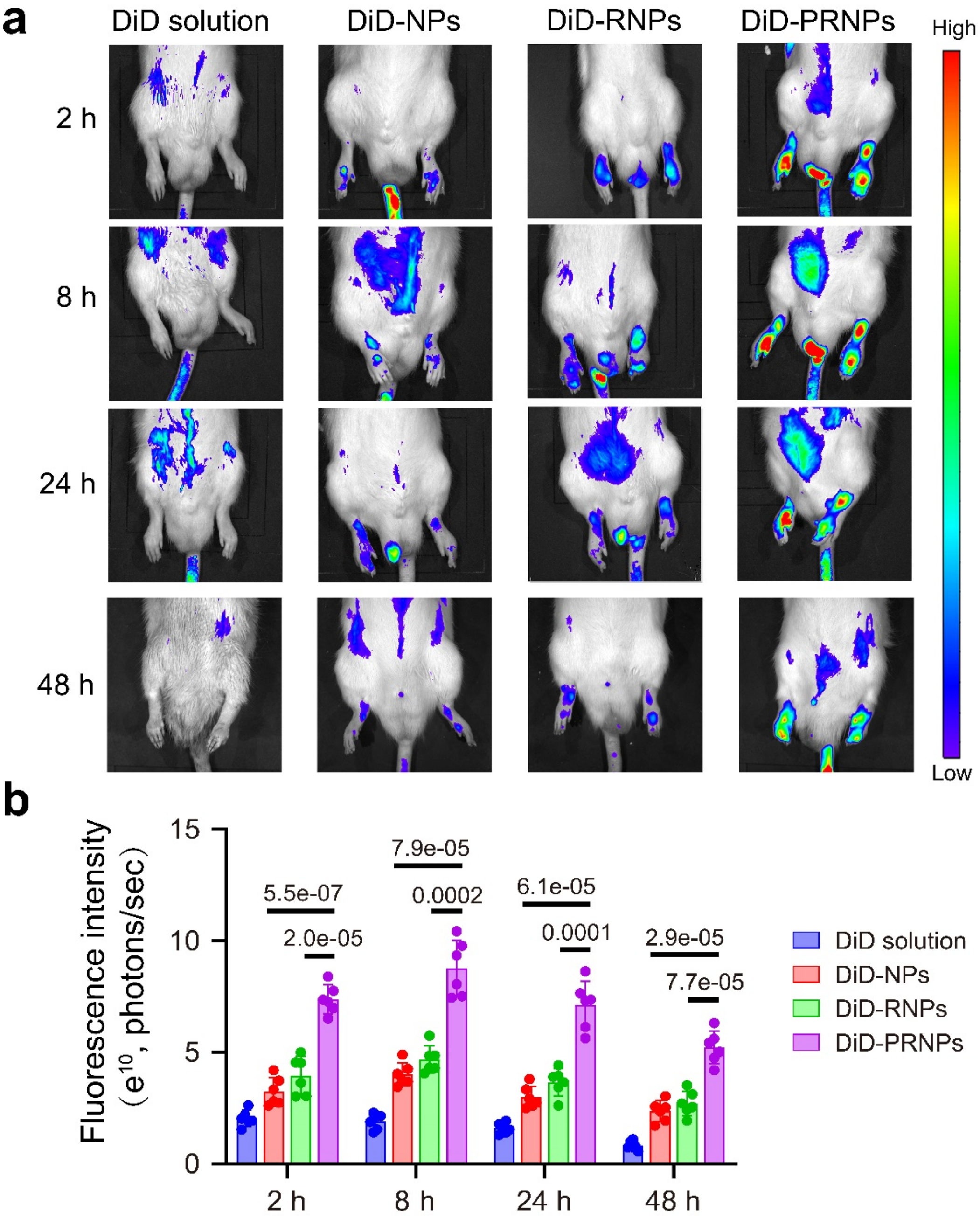

Supplementary Figure 8: Inflamed joint distribution of PRNPs in AIA rats with advanced arthritis. (a) *In vivo* DiD fluorescence images showing the arthritic joint distribution of free DiD, DiD-loaded NPs, DiD-loaded RNPs and DiD-loaded PRNPs in the late stage of AIA rats at 2 h, 8 h, 24 h and 48 h post-injection (*n* = 3 independent animals). (b) The statistical graphs of the fluorescence intensity of arthritic joints based on the semi-quantitative analysis of the *in vivo* fluorescence images of rats (*n* = 6 inflamed joints from 3 independent animals) after *i.v.* administration of free DiD or DiD-labeled nanoparticles. Data represent mean ± SD. Statistical significance was determined by two-sided Student's t-test. DiD, 1,1′-dioctadecyl-3,3,3′,3′-

tetramethyl indodicarbocyanine, 4-chlorobenzenesulfonate salt; DiD-NPs, DiD labeled poly (D, L-lactide-co-glycolide) (PLGA) nanoparticles; DiD-RNPs, DiD-labeled RGD peptide-modified PLGA nanoparticles; DiD-PRNPs, DiD-labeled matrix metalloproteinase 9 (MMP9)-cleavable polyethylene glycol (PEG)- and RGD peptide-modified PLGA nanoparticles.

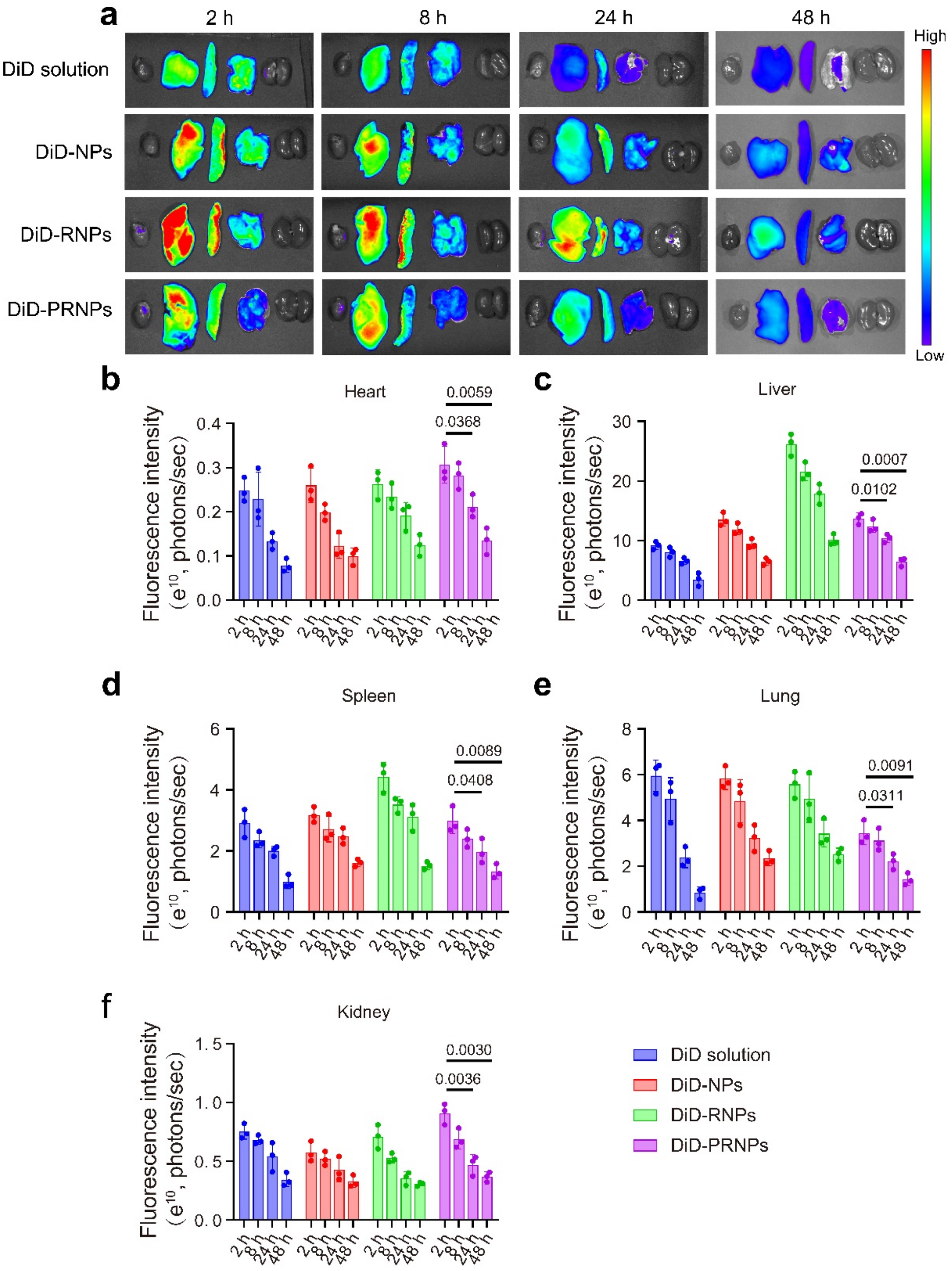

Supplementary Figure 9: Tissue distribution of PRNPs in AIA rats with advanced arthritis.

(a) *Ex vivo* DiD fluorescence images in major organs of AIA rats with advanced arthritis at 2 h, 8 h, 24 h and 48 h post-injection with free DiD, or DiD-labeled NPs, RNPs or PRNPs (*n* = 3 independent animals). (b-f) The statistical graphs of the fluorescence intensity of organs based on the semi-quantitative analysis of the *ex vivo* fluorescence images after *i.v.* administration of free DiD or DiD-labeled nanoparticles. Data represent mean ± SD (*n* = 3 independent animals). Statistical significance was determined by two-sided Student's t-test. DiD, 1,1′-dioctadecyl-3,3,3′,3′-tetramethyl indodicarbocyanine, 4-chlorobenzenesulfonate salt; DiD-NPs, DiD labeled poly (D, L-lactide-co-glycolide) (PLGA) nanoparticles; DiD-RNPs, DiD-labeled RGD peptide-modified PLGA nanoparticles; DiD-PRNPs, DiD-labeled matrix metalloproteinase 9 (MMP9)-cleavable polyethylene glycol (PEG)- and RGD peptide-modified PLGA nanoparticles.

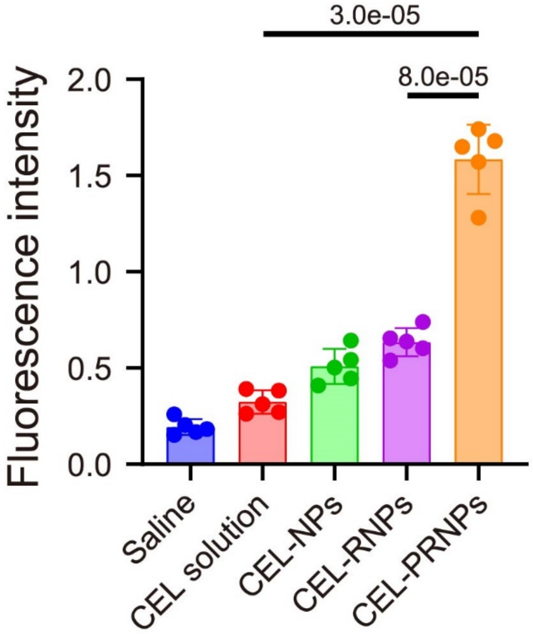

Supplementary Figure 10: Quantitative analysis for the TUNEL staining. Quantitative analysis for the immunofluorescence of TUNEL staining in arthritic joints of AIA rats with late-stage arthritis after receiving the indicated treatment. Data represent mean ± SD (*n* = 5 independent animals). Statistical significance was determined by two-sided Student's t-test. CEL, celastrol; CEL-NPs, CEL-loaded poly (D, L-lactide-co-glycolide) (PLGA) nanoparticles; CEL-RNPs, CEL-loaded RGD peptide-modified PLGA nanoparticles; CEL-PRNPs, CEL-loaded matrix metalloproteinase 9 (MMP9)-cleavable polyethylene glycol (PEG)- and RGD peptide-modified PLGA nanoparticles.

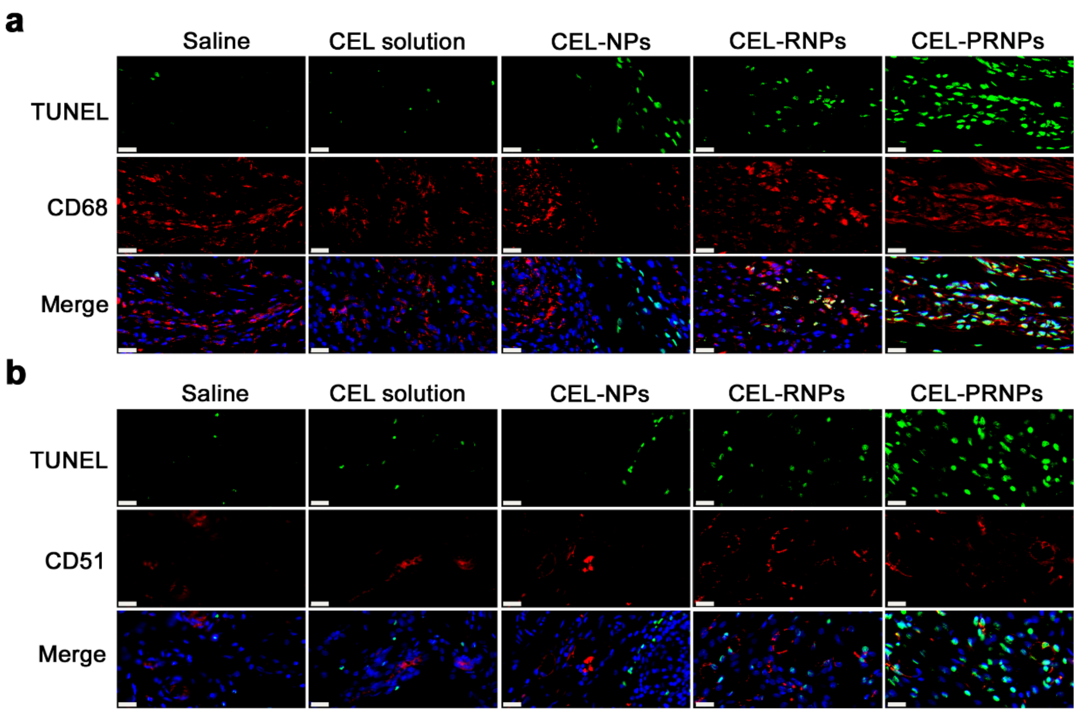

Supplementary Figure 11: TUNEL immunofluorescence staining in ankle joints from AIA rats receiving the indicated treatment. Macrophages and OCs were determined by immunofluorescence analysis of CD68 and CD51 (red fluorescence), respectively. (Scale bar = 20 μm), (*n* = 3 independent animals). CEL, celastrol; CEL-NPs, CEL-loaded poly (D, L-lactide-co-glycolide) (PLGA) nanoparticles; CEL-RNPs, CEL-loaded RGD peptide-modified PLGA nanoparticles; CEL-PRNPs, CEL-loaded matrix metalloproteinase 9 (MMP9)-cleavable polyethylene glycol (PEG)- and RGD peptide-modified PLGA nanoparticles; TUNEL, TdT-mediated dUTP nick end labeling.

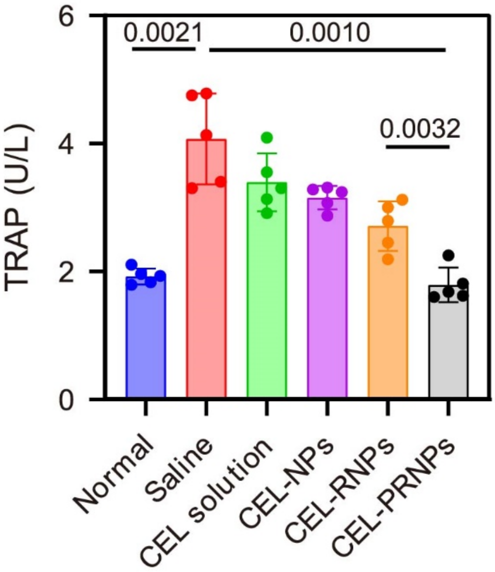

Supplementary Figure 12: TRAP level in the blood from AIA rats with late-stage arthritis after treatment. Data represent mean ± SD (*n* = 5 independent animals). Statistical significance was determined by two-sided Student's t-test. CEL, celastrol; CEL-NPs, CEL-loaded poly (D, L-lactide-co-glycolide) (PLGA) nanoparticles; CEL-RNPs, CEL-loaded RGD peptide-modified PLGA nanoparticles; CEL-PRNPs, CEL-loaded matrix metalloproteinase 9 (MMP9)-cleavable polyethylene glycol (PEG)- and RGD peptide-modified PLGA nanoparticles; TRAP, tartrate-resistant acid phosphatase.

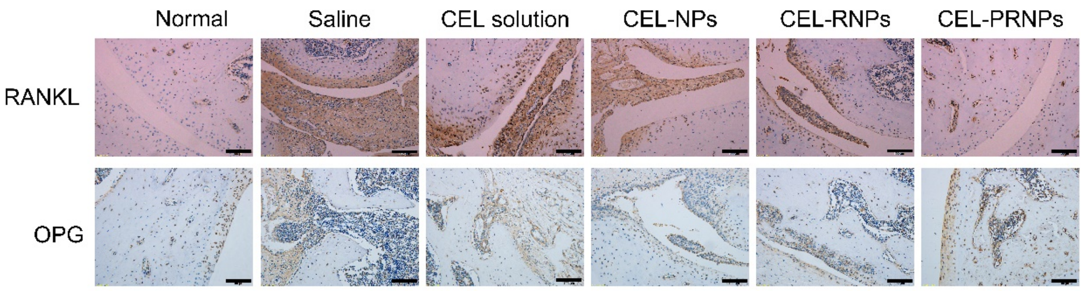

Supplementary Figure 13: Detection of RANKL and OPG expression level in arthritic joints. Arthritic joints of different groups were stained with RANKL and OPG antibodies, respectively (Scale bar = 100 μm) (*n* = 5 independent animals). CEL, celastrol; CEL-NPs, CEL-loaded poly (D, L-lactide-co-glycolide) (PLGA) nanoparticles; CEL-RNPs, CEL-loaded RGD peptide-modified PLGA nanoparticles; CEL-PRNPs, CEL-loaded matrix metalloproteinase 9 (MMP9)-cleavable polyethylene glycol (PEG)- and RGD peptide-modified PLGA nanoparticles; RANKL, receptor of activator of NF-kB ligand; OPG, osteoprotegerin.

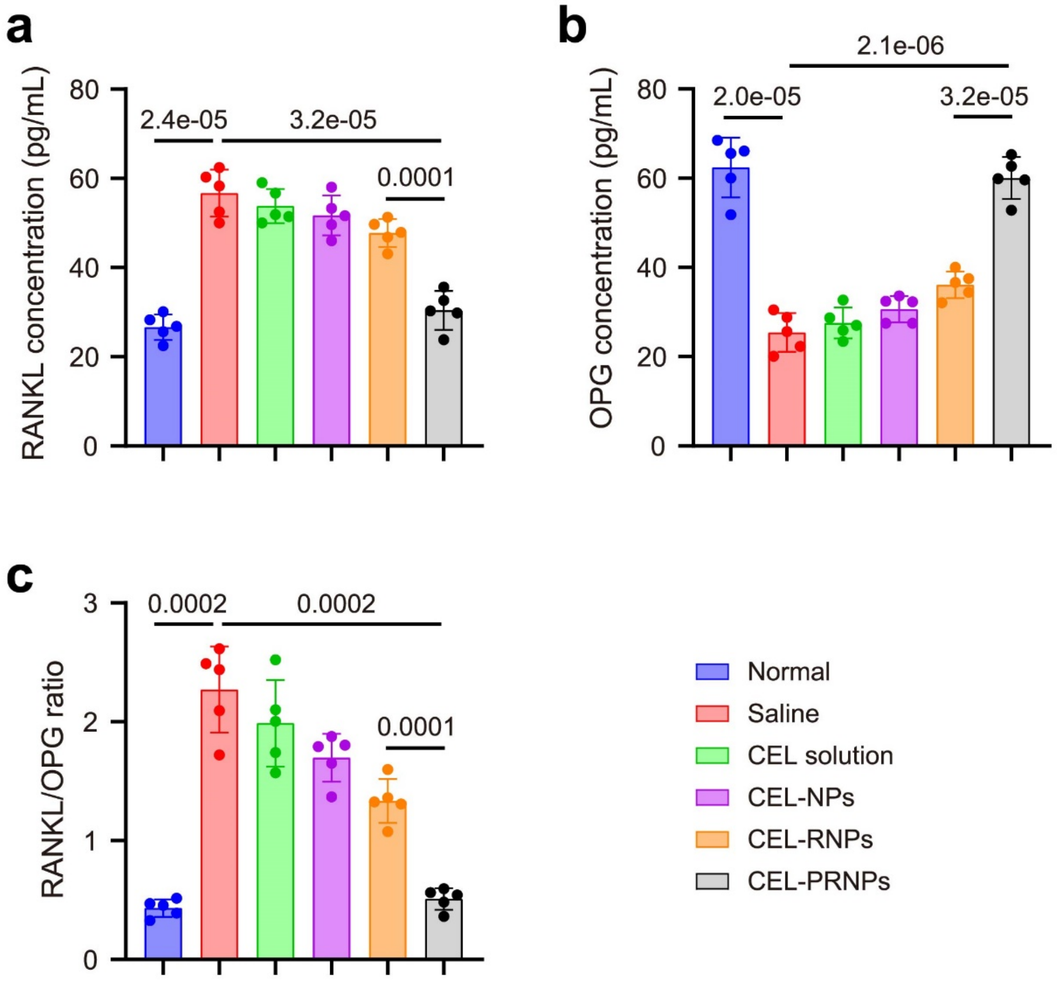

Supplementary Figure 14: Blood level of RANKL, OPG and RANK/OPG ratio in AIA rats after treatment. (a) The blood level of RANKL in AIA rats with late-stage arthritis after receiving the indicated treatment. Data represent mean ± SD (*n* = 5 independent animals). Statistical significance was determined by two-sided Student's t-test. (b) The blood level of OPG in AIA rats with late-stage arthritis after receiving the indicated treatment. Data represent mean ± SD (*n* = 5 independent animals). Statistical significance was determined by two-sided Student's t-test. (c) The blood level of RANK/OPG ratio in AIA rats with late-stage arthritis after receiving the indicated treatment. Data represent mean ± SD (*n* = 5 independent animals). Statistical significance was determined by two-sided Student's t-test.CEL, celastrol; CEL-NPs, CEL-loaded poly (D, L-lactide-co-glycolide) (PLGA) nanoparticles; CEL-RNPs, CEL-loaded RGD peptide-modified PLGA nanoparticles; CEL-PRNPs, CEL-loaded matrix metalloproteinase 9 (MMP9)-cleavable polyethylene glycol (PEG)- and RGD peptide-modified PLGA nanoparticles; RANKL, receptor of

activator of NF-kB ligand; OPG, osteoprotegerin.

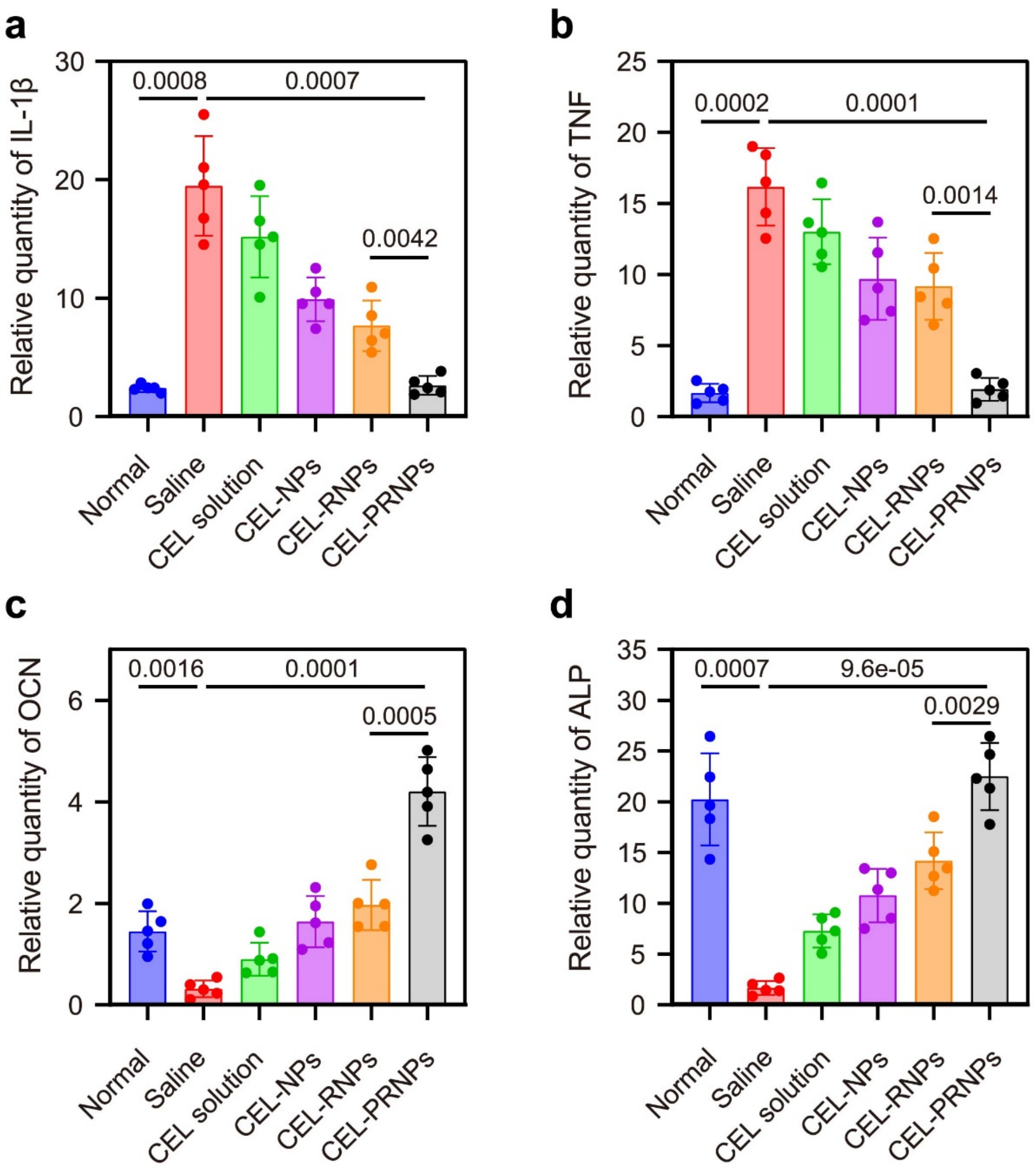

Supplementary Figure 15: Relative quantity of IL-1β, TNF, OCN and ALP in arthritic joints. (a) The relative quantity of IL-1β in arthritic joints in different groups. Data represent mean ± SD (*n* = 5 independent animals). Statistical significance was determined by two-sided Student's t-test. (b) The relative quantity of TNF in arthritic joints in different groups. Data represent mean ± SD (*n* = 5 independent animals). Statistical significance was determined by two-sided Student's t-test. (c) The relative quantity of OCN in arthritic joints in different groups. Data represent mean ± SD (*n* = 5 independent animals). Statistical significance was determined by two-sided Student's t-test. (d) The relative quantity of ALP in arthritic joints in different groups. Data represent mean ± SD (*n* = 5

independent animals). Statistical significance was determined by two-sided Student's t-test. CEL, celastrol; CEL-NPs, CEL-loaded poly (D, L-lactide-co-glycolide) (PLGA) nanoparticles; CEL-RNPs, CEL-loaded RGD peptide-modified PLGA nanoparticles; CEL-PRNPs, CEL-loaded matrix metalloproteinase 9 (MMP9)-cleavable polyethylene glycol (PEG)- and RGD peptide-modified PLGA nanoparticles; IL-1β, interleukin-1 β; TNF, tumor necrosis factor; OCN, osteocalcin; ALP, alkaline phosphatase.

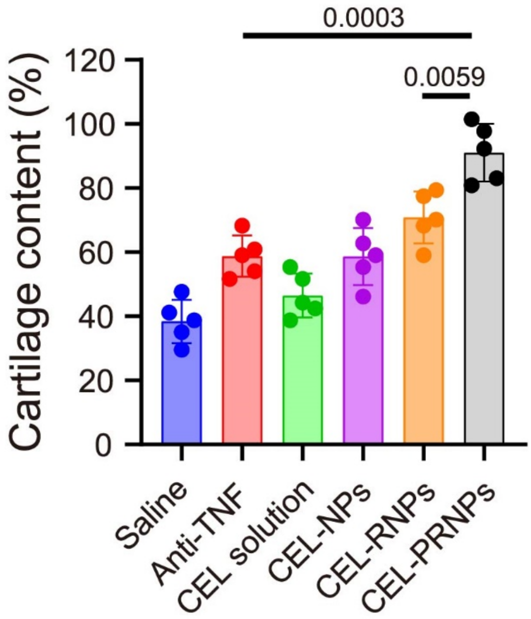

Supplementary Figure 16: Quantitative analysis for the safranin O-positive area of ankle joints of AIA rats with advanced arthritis after treatment. Data represent mean ± SD (n = 5 independent animals). Statistical significance was determined by two-sided Student's t-test. Anti-TNF, anti-TNF (tumor necrosis factor) antibody; CEL, celastrol; CEL-NPs, CEL-loaded poly (D, L-lactide-co-glycolide) (PLGA) nanoparticles; CEL-RNPs, CEL-loaded RGD peptide-modified PLGA nanoparticles; CEL-PRNPs, CEL-loaded matrix metalloproteinase 9 (MMP9)-cleavable polyethylene glycol (PEG)- and RGD peptide-modified PLGA nanoparticles.

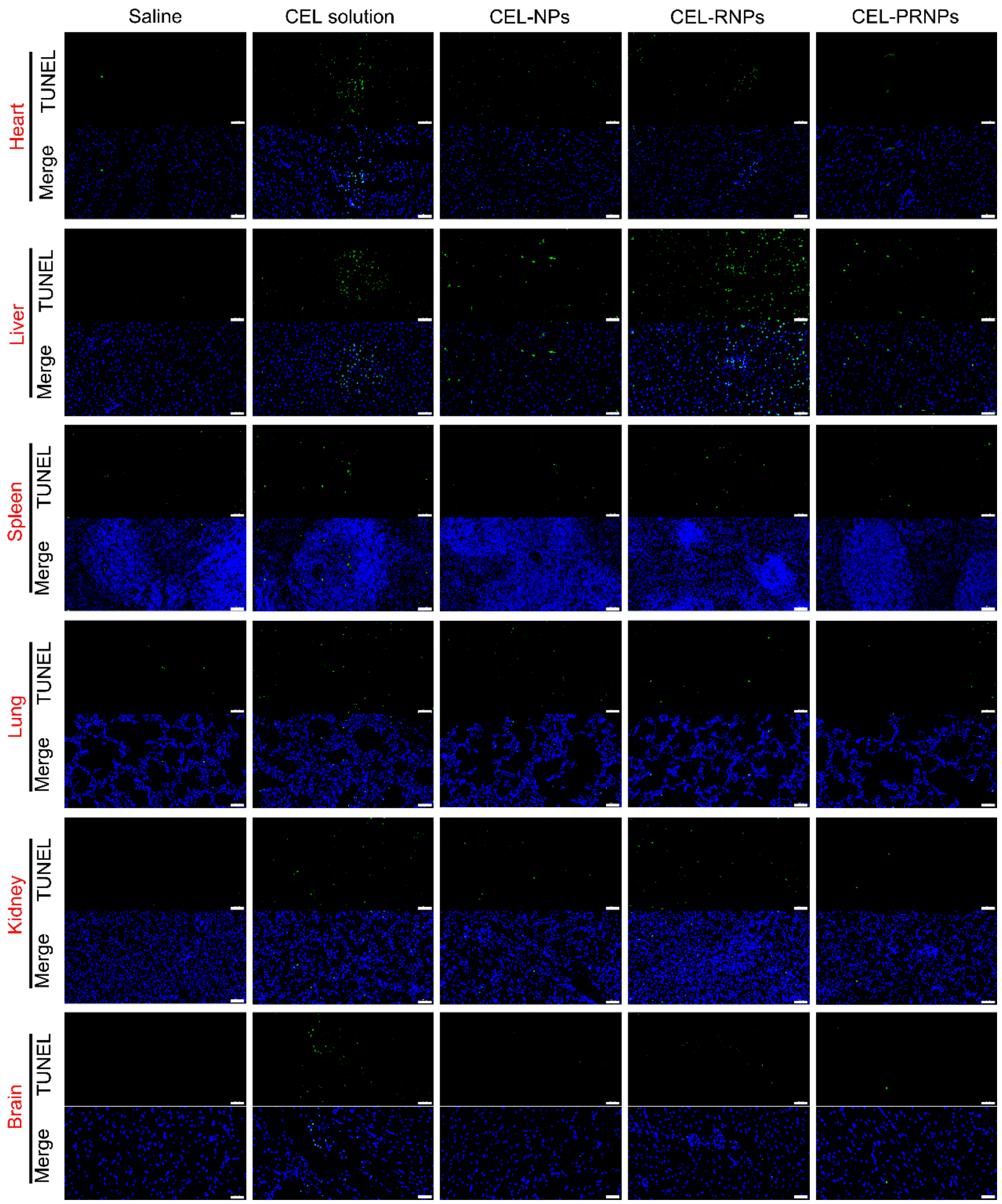

Supplementary Figure 17: TUNEL immunofluorescence staining of major organs. 2 days after rats receiving the indicated treatment, major organs (heart, liver, spleen, lung, kidney and brain) were collected to conduct TUMEL immunofluorescence staining. The fluorescent of TUNEL staining was observed with a laser scanning confocal microscope (Scale bar = 50 μm) (*n* = 3 independent animals). CEL, celastrol; CEL-NPs, CEL-loaded poly (D, L-lactide-co-glycolide) (PLGA) nanoparticles; CEL-RNPs, CEL-loaded RGD peptide-modified PLGA nanoparticles; CEL-PRNPs, CEL-loaded matrix metalloproteinase 9 (MMP9)-cleavable polyethylene glycol (PEG)- and RGD peptide-modified PLGA nanoparticles; TUNEL, TdT-mediated dUTP nick end labeling.

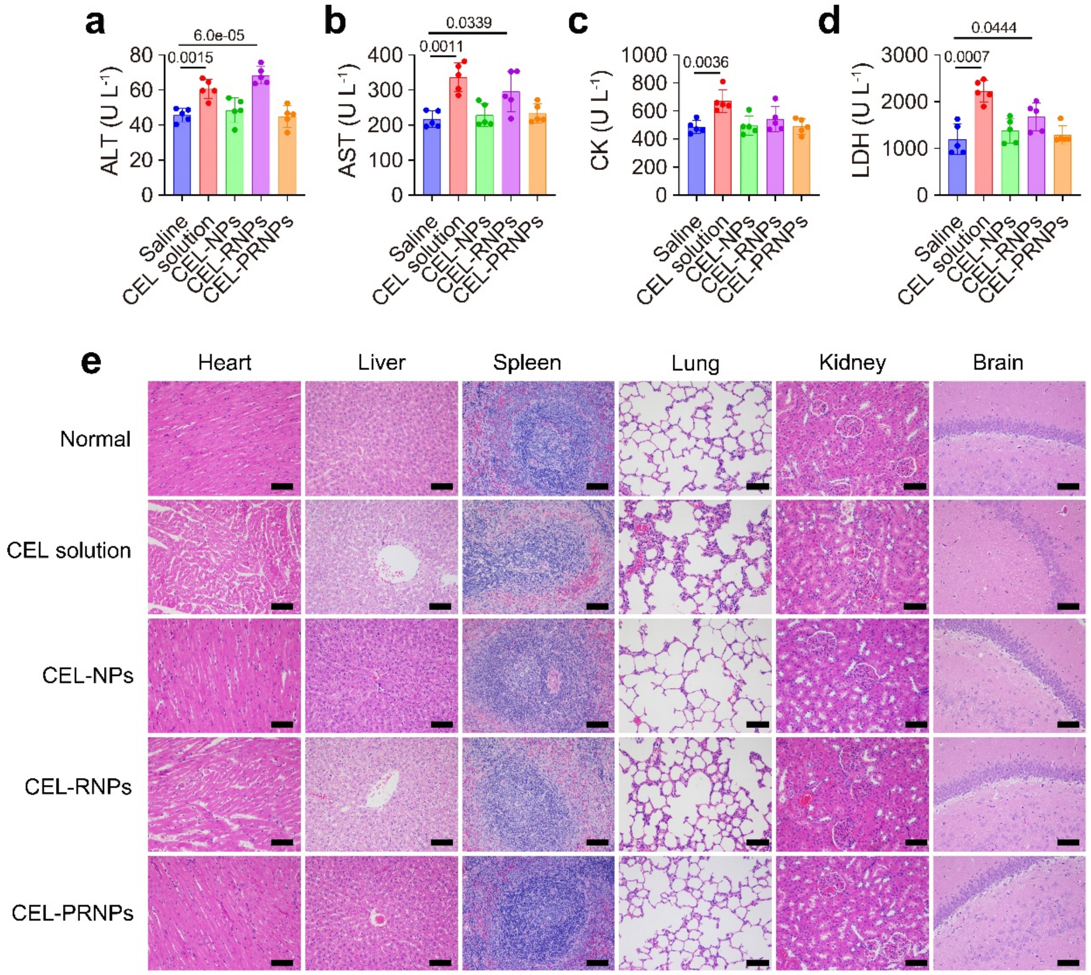

Supplementary Figure 18: Safety evaluation. (a-d) The serum level of ALT (a), AST (b), CK (c) and LDH (d) in rats receiving the indicated treatment. Data represent mean ± SD (*n* = 5 independent animals). Statistical significance was determined by two-sided Student's t-test. (e) Hematoxylin and eosin (H&E) staining of the major organs (heart, liver, spleen, lung, kidney and brain) were processed 2 days after rats receiving the indicated treatment. (Scale bar = 100 μm) (*n* = 5 independent animals). ALT, alanine aminotransferase; AST, aspartate aminotransferase; CK, creatine kinase; LDH, lactic dehydrogenase; CEL, celastrol; CEL-NPs, CEL-loaded poly (D, L-lactide-co-glycolide) (PLGA) nanoparticles; CEL-RNPs, CEL-loaded RGD peptide-modified PLGA nanoparticles; CEL-PRNPs, CEL-loaded matrix metalloproteinase 9 (MMP9)-cleavable polyethylene glycol (PEG)- and RGD peptide-modified PLGA nanoparticles.

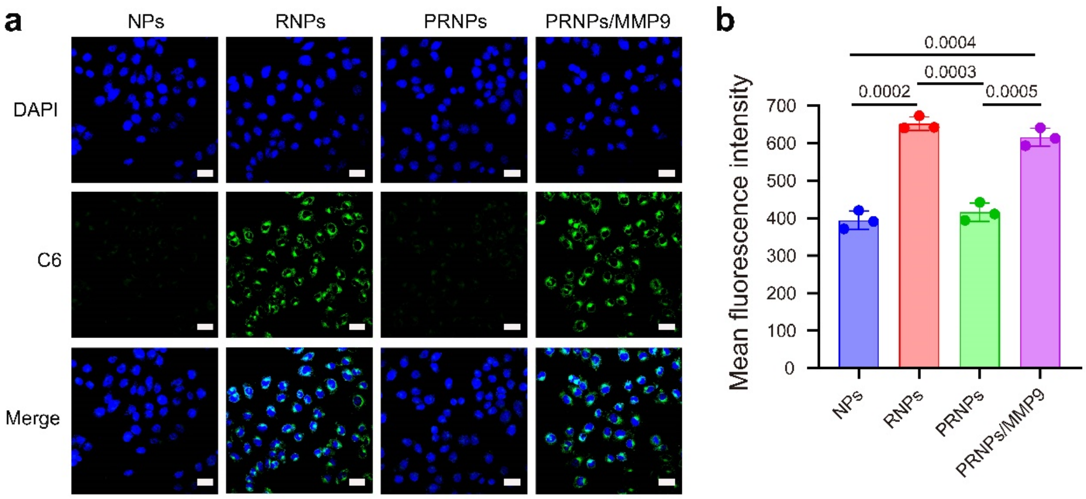

Supplementary Figure 19: Cellular uptake of PRNPs on TNF-activated HUVECs. (a) Confocal images (Scale bar = 50 µm) of the cellular uptake of C6-loaded NPs, C6-loaded RNPs, and C6-loaded PRNPs in TNF-activated HUVECs (*n* = 3 independent samples). (b) Quantitative analysis of the cellular uptake of C6-NPs, C6-RNPs and C6-PRNPs in TNF-activated HUVECs after 1-h incubation at the C6 concentration of 50 ng/mL. Data represent mean ± SD (*n* = 3 independent samples). Statistical significance was determined by two-sided Student's t-test. C6, coumarin 6; DAPI, 2-(4-Amidinophenyl)-6-indolecarbamidine dihydrochloride; MMP9, matrix metalloproteinase 9; NPs, poly (D, L-lactide-co-glycolide) (PLGA) nanoparticles; RNPs, RGD peptide-modified PLGA nanoparticles; PRNPs, MMP9-cleavable polyethylene glycol (PEG)- and RGD peptide-modified PLGA nanoparticles.

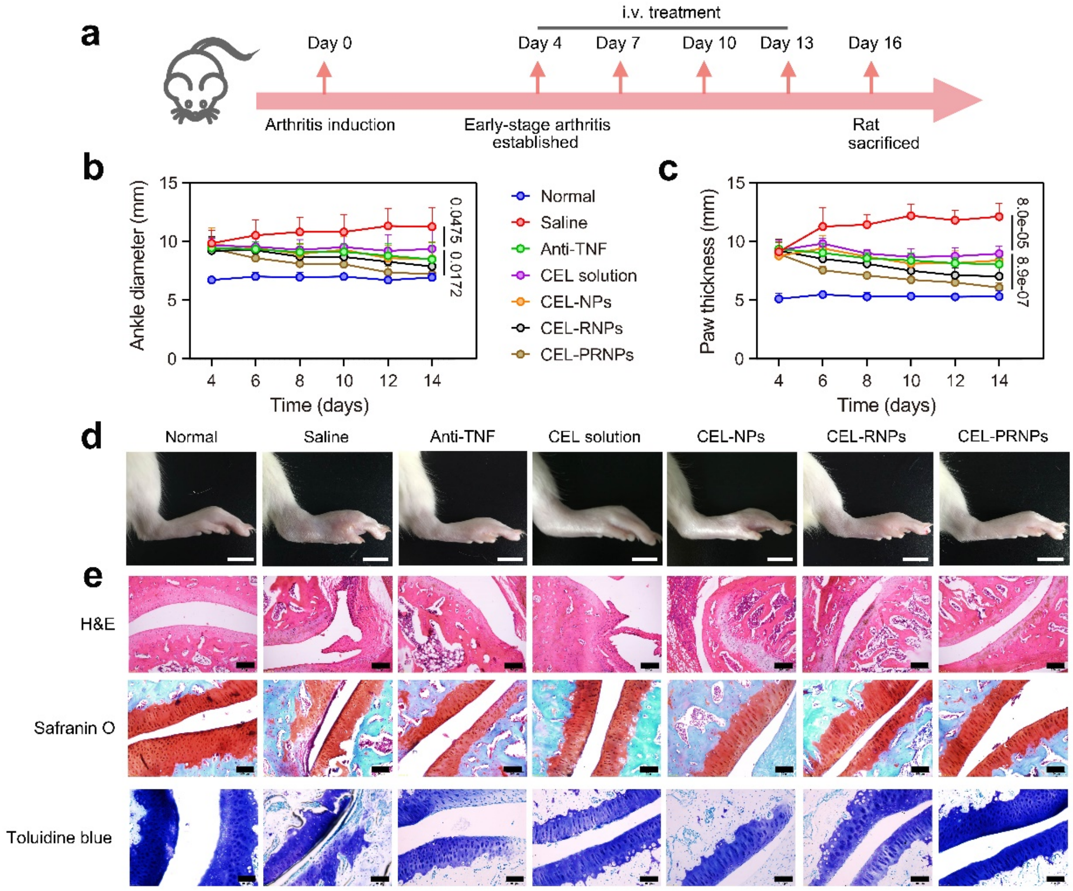

Supplementary Figure 20: CEL-PRNPs alleviating synovial inflammation and inhibiting cartilage damages in the early stage of AIA rat model. (a) The schematic illustration of CEL-PRNPs treatment. (b, c) Paw thickness (b) and ankle diameter (c) of AIA rats were recorded every other day during the treatment period. Data represent mean ± SD (*n* = 7 independent animals). Statistical significance was determined by two-sided Student's t-test. (d) Representative photographs of the hindlimbs at the endpoint of the experiment from different treatment groups (Scale bar = 10 mm); (e) Histopathology evaluation of the ankle joints was identified using H&E (Scale bar = 200 μm), Safranin-O and toluidine blue staining (Scale bar = 100 μm) (*n* = 5 independent animals). Anti-TNF, anti-TNF (tumor necrosis factor) antibody; CEL, celastrol; CEL-NPs, CEL-loaded poly (D, L-lactide-co-glycolide) (PLGA) nanoparticles; CEL-RNPs, CEL-loaded RGD peptide-modified PLGA nanoparticles; CEL-PRNPs, CEL-loaded matrix metalloproteinase 9 (MMP9)-cleavable polyethylene glycol (PEG)- and RGD peptide-modified PLGA nanoparticles; H&E, hematoxylin-eosin.

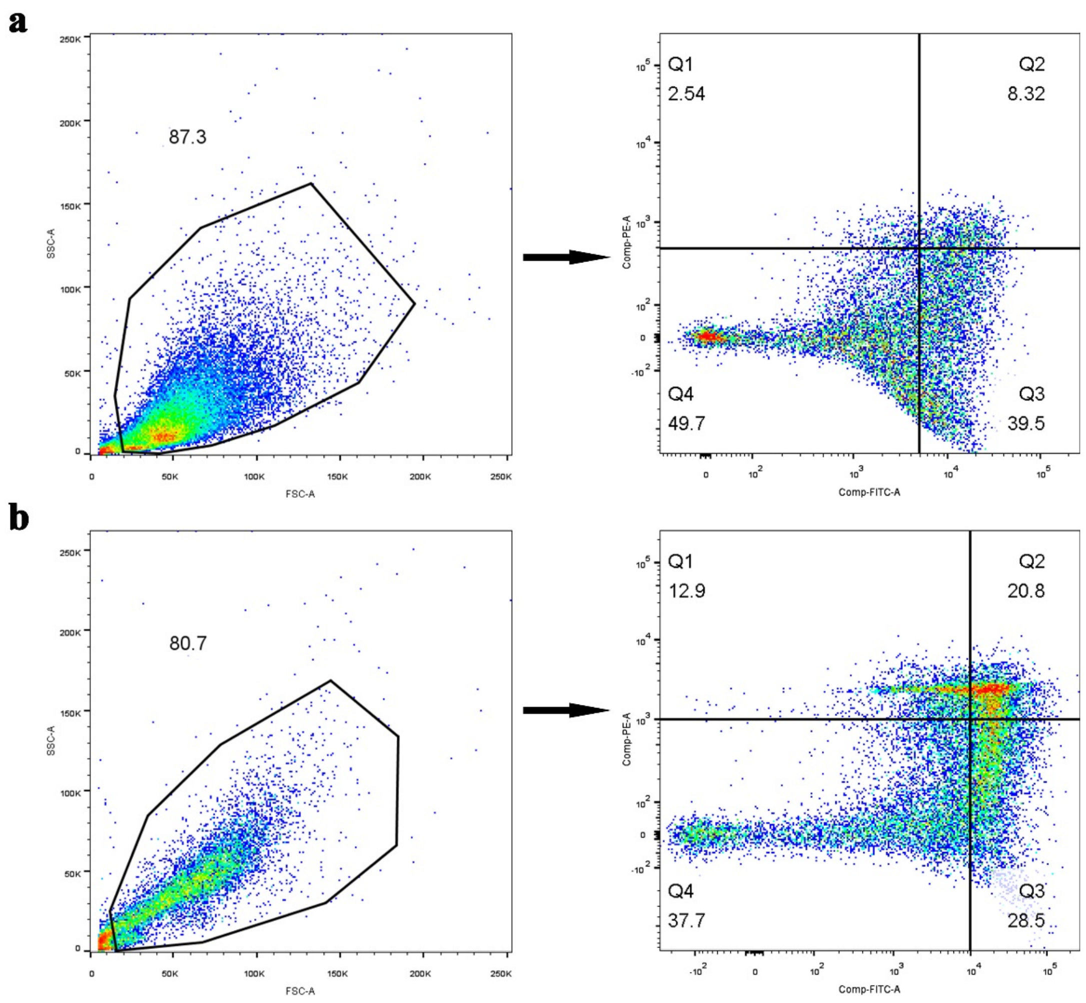

Supplementary Figure 21: Flow cytometry gating strategy for cell apoptosis assay. (a) Flow cytometry gating strategy for osteoclasts apoptosis assay. A forward/side scatter dot plot (left) was used to gate the main cell population, and the gated cells were then analyzed by FITC-Annexin V and PI staining patterns in a FL1/FL2 plot (right). The proportion of cells undergoing viable (double-negative for FITC-annexin V and PI), early apoptosis (positive for FITC-annexin V), late apoptosis (double-positive for FITC- annexin V and PI), and necrosis (positive for PI) can be found in quadrants Q4, Q3, Q2 and Q1, respectively. In our study, the total percentage of apoptosis induction is defined as the sum of early apoptotic and late apoptotic cells. (b) Flow cytometry gating strategy for macrophages apoptosis assay. A forward/side scatter dot plot (left) was used to gate the main cell population, and the gated cells were then analyzed by FITC-Annexin V and PI staining patterns in a FL1/FL2 plot (right). The proportion of cells undergoing viable (double-negative for FITC-annexin V and PI), early apoptosis (positive for FITC-annexin V), late apoptosis (double-positive

for FITC- annexin V and PI), and necrosis (positive for PI) can be found in quadrants Q4, Q3, Q2 and Q1, respectively. In our study, the total percentage of apoptosis induction is defined as the sum of early apoptotic and late apoptotic cells.

Supplementary table 1. Characterization of CEL-NPs, CEL-RNPs and CEL-PRNPs. Data represent mean ± SD (*n* = 3 independent samples).

	Particle size (nm)	PDI	Zeta potential (mV)	EE (%)
CEL-NPs	155.7 ± 4.9	0.102 ± 0.027	-2.5 ± 0.4	88.5 ± 4.2
CEL-RNPs	154.1 ± 4.6	0.116 ± 0.043	-3.2 ± 0.6	90.6 ± 5.8
CEL-PRNPs	162.2 ± 6.6	0.167 ± 0.041	-5.3 ± 0.4	87.4 ± 6.1
