# Supplementary material for: Targeted apoptosis of macrophages and osteoclasts in arthritic joints is effective against advanced inflammatory arthritis
Source: Nat Commun. 2021 Apr 12;12:2174. doi: 10.1038/s41467-021-22454-z (PMC8042091; doi:10.1038/s41467-021-22454-z)
Supplement: Supplementary file 3 — Reporting Summary [file 41467_2021_22454_MOESM3_ESM.pdf]

nature research

Corresponding author(s): Tao Gong

Last updated by author(s): Feb 28, 2021

# Reporting Summary

Nature Research wishes to improve the reproducibility of the work that we publish. This form provides structure for consistency and transparency in reporting. For further information on Nature Research policies, see our Editorial Policies and the Editorial Policy Checklist.

## Statistics

For all statistical analyses, confirm that the following items are present in the figure legend, table legend, main text, or Methods section.

| n/a | Confirmed | |
|---|---|---|
| [ ] | [x] | The exact sample size (*n*) for each experimental group/condition, given as a discrete number and unit of measurement |
| [ ] | [x] | A statement on whether measurements were taken from distinct samples or whether the same sample was measured repeatedly |
| [ ] | [x] | The statistical test(s) used AND whether they are one- or two-sided<br>*Only common tests should be described solely by name; describe more complex techniques in the Methods section.* |
| [x] | [ ] | A description of all covariates tested |
| [x] | [ ] | A description of any assumptions or corrections, such as tests of normality and adjustment for multiple comparisons |
| [ ] | [x] | A full description of the statistical parameters including central tendency (e.g. means) or other basic estimates (e.g. regression coefficient) AND variation (e.g. standard deviation) or associated estimates of uncertainty (e.g. confidence intervals) |
| [ ] | [x] | For null hypothesis testing, the test statistic (e.g. *F*, *t*, *r*) with confidence intervals, effect sizes, degrees of freedom and *P* value noted<br>*Give P values as exact values whenever suitable.* |
| [x] | [ ] | For Bayesian analysis, information on the choice of priors and Markov chain Monte Carlo settings |
| [x] | [ ] | For hierarchical and complex designs, identification of the appropriate level for tests and full reporting of outcomes |
| [x] | [ ] | Estimates of effect sizes (e.g. Cohen's *d*, Pearson's *r*), indicating how they were calculated |

*Our web collection on statistics for biologists contains articles on many of the points above.*

## Software and code

Policy information about availability of computer code

| | |
|---|---|
| Data collection | No stand-alone or custom software was used. |
| Data analysis | Softwares used in analysis include FlowJo V10, GraphPad Prism 8, Image J1.52, Microsoft Excel2010. |

For manuscripts utilizing custom algorithms or software that are central to the research but not yet described in published literature, software must be made available to editors and reviewers. We strongly encourage code deposition in a community repository (e.g. GitHub). See the Nature Research guidelines for submitting code & software for further information.

## Data

Policy information about availability of data

All manuscripts must include a data availability statement. This statement should provide the following information, where applicable:

- Accession codes, unique identifiers, or web links for publicly available datasets
- A list of figures that have associated raw data
- A description of any restrictions on data availability

The source data underlying Figures 1b, c, 2d, 3d-g, 4b-f, 5d-f, 7c, 8b, c and 9c-g and Supplementary Figure 3a, b, 7b, 8b, 9b-f, 10, 12, 14a-c, 15a-d, 16, 18a-d, 19b and 20b, c and Supplementary Table 1 are provided as a Source Data file. All relevant data are also available in the Article, Supplementary Information file of available from the corresponding author upon request.

# Field-specific reporting

Please select the one below that is the best fit for your research. If you are not sure, read the appropriate sections before making your selection.

☒ Life sciences ☐ Behavioural & social sciences ☐ Ecological, evolutionary & environmental sciences

For a reference copy of the document with all sections, see nature.com/documents/nr-reporting-summary-flat.pdf

# Life sciences study design

All studies must disclose on these points even when the disclosure is negative.

| | |
|---|---|
| Sample size | No sample size calculation was performed. Sample size was determined to be adequate based on the magnitude and consistency of measurable differences between groups. In addition, the sample sizes of this study were determined on the basis of similar published studies. In vitro experiments, the sample size for each group was 3.The in vivo efficacy studies were performed with 7 rats per group. Details regarding sample size of all experiments are provided in the Methods section and figure legends. |
| Data exclusions | No data were excluded from the analyses. |
| Replication | Reproducibility was consistent across different biological replicates of experiments. |
| Randomization | Samples were randomly allocated to corresponding experimental groups. Organisms were cultured and maintained in the same environment and randomly allocated to each group. |
| Blinding | The investigators were not blinded to group allocation during data collection except some data (HE, immunofluorescence staining). Before the experiment were performed, we have to check the study procedure and analysis methods were correctly done. However, the treatment efficacy was apparent in both quantification and representative images of outcomes |

# Reporting for specific materials, systems and methods

We require information from authors about some types of materials, experimental systems and methods used in many studies. Here, indicate whether each material, system or method listed is relevant to your study. If you are not sure if a list item applies to your research, read the appropriate section before selecting a response.

## Materials & experimental systems

| n/a | Involved in the study | |
|---|---|---|
| ☐ | ☒ | Antibodies |
| ☐ | ☒ | Eukaryotic cell lines |
| ☒ | ☐ | Palaeontology and archaeology |
| ☐ | ☒ | Animals and other organisms |
| ☐ | ☒ | Human research participants |
| ☒ | ☐ | Clinical data |
| ☒ | ☐ | Dual use research of concern |

## Methods

| n/a | Involved in the study | |
|---|---|---|
| ☒ | ☐ | ChIP-seq |
| ☐ | ☒ | Flow cytometry |
| ☒ | ☐ | MRI-based neuroimaging |

## Antibodies

| | |
|---|---|
| Antibodies used | Anti-CD68 (Cat#ab125212, 1:500), Anti-CD51 (Cat#ab179475, 1:500), Anti-MMP9 (Cat#ab76003, 1:1000), Anti-Osteoprotegerin (Cat#ab203061, 1:200), Anti-IL-1β (Cat#ab205924, 1:200), Anti-RANKL(Cat#ab239607, 1:100), Anti-Osteocalcin (Cat#ab13420, 1:200), Anti-TNF (Cat#ab220210, 1:100) and Anti-ALP (Cat#ab224335, 1:200) were purchased from Abcam. |
| Validation | All primary antibodies were verified to be validated by the manufacturer for species specificity and the application used. |

## Eukaryotic cell lines

Policy information about cell lines

| | |
|---|---|
| Cell line source(s) | Bone marrow cells were isolated from the tibiae of C57BL/J mice. Bone marrow cells were cultured with 30 ng/mL of M-CSF for 2 days to obtain bone marrow macrophages (BMMs). These BMMs were cultured in the presence of 100 ng /mL of RANKL and 30 ng/mL of M-CSF for 4 days to finally generate osteoclasts (OCs). Human primary synovial macrophages were isolated from synovial tissue specimens of patients with late-stage rheumatic arthritis (RA) undergoing joint replacement surgery. Peripheral blood mononuclear cells (PBMCs) from RA patients were cultured in the presence of 100 ng /mL of RANKL and 30 ng/mL of M-CSF for 4 days to generate RA patients derived OCs. Human umbilical vein endothelial cells (HUVECs) were purchased from Chinese Academy of Sciences Cell Bank for Type Culture Collection (Shanghai, China). |

| | |
|---|---|
| Authentication | Osteoclasts were multinuclear under microscopic examination and they could be stained red by TRAP. Details regarding osteoclasts authentication are provided in the Methods and Surpporting Information section. No further authentication was conducted on HUVECs. |
| Mycoplasma contamination | All cell lines were tested negative for Mycoplasm contamination. |
| Commonly misidentified lines (See ICLAC register) | No commonly misidentified lines were used in this study. |

## Animals and other organisms

Policy information about studies involving animals; ARRIVE guidelines recommended for reporting animal research

| | |
|---|---|
| Laboratory animals | Healthy male Wistar rats (5-weeks-old, 200 ± 20 g) and male C57BL/J mice (6-weeks-old, 20 ± 2 g) were obtained from Chengdu Dashuo Experimental Animal Co., Ltd (Chengdu, China). Rats were randomized before the experiment. |
| Wild animals | No wild animals were used in this study. |
| Field-collected samples | No field-collected samples were used in the study. |
| Ethics oversight | All animal studies were conducted according to the requirements of the national act on the use of experimental animals (China) and in compliance with guidelines evaluated and approved by the Animal Ethics Committee of Sichuan University. |

Note that full information on the approval of the study protocol must also be provided in the manuscript.

## Human research participants

Policy information about studies involving human research participants

| | |
|---|---|
| Population characteristics | To confirm the potential of the developed drug delivery vehicle targeting human osteoclasts and macrophages in inflamed joints, peripheral blood samples and synovial tissues were obtained from rheumatoid arthritis (RA) patients. Three female patients aged from 50 to 74, were diagnosed with late-stage RA (according to the American College of Rheumatology criteria) . All three RA patients were in the need of undergoing joint replacement surgery. |
| Recruitment | Participants were not directly recruited into the study. The blood samples and synovial tissues from three RA patients were collected only for obtaining human OCs and synovial macrophages. |
| Ethics oversight | The collection of peripheral blood samples and synovial tissues from RA patients were approved by Ethics Committee of the Xiangya Hospital of Central South University. |

Note that full information on the approval of the study protocol must also be provided in the manuscript.

## Flow Cytometry

### Plots

Confirm that:

- [x] The axis labels state the marker and fluorochrome used (e.g. CD4-FITC).
- [x] The axis scales are clearly visible. Include numbers along axes only for bottom left plot of group (a 'group' is an analysis of identical markers).
- [x] All plots are contour plots with outliers or pseudocolor plots.
- [x] A numerical value for number of cells or percentage (with statistics) is provided.

### Methodology

| | |
|---|---|
| Sample preparation | Bone marrow cells isolated from the tibiae of C57BL/J mice were cultured with 30 ng/mL of M-CSF for 2 days and used as bone marrow macrophages (BMMs). To generate OCs, these BMMs were cultured in the presence of 100 ng /mL of RANKL and 30 ng/mL of M-CSF for 4 days. To obtain activated macrophages, BMMs were treated with 10 ng/mL of LPS for 48 h. All cells were cultured in RPMI-1640 medium containing 10% FBS and 100 U/mL of penicillin-streptomycin under 5% CO2 at 37 ° C. Details on cellular uptake and apoptosis studies are provide in Methods section. |
| Instrument | BD FACSCelestaTM flow cytometer. |
| Software | FlowJo V10. |
| Cell population abundance | o cell population was sorted. |
| Gating strategy | A forward/side scatter (FS/SS) dot plot was used to gate the main cell population, and the gated cells were then analyzed by FITC-Annexin V and PI staining patterns in a FITC/PE plot . the proportion of cells undergoing viable (double-negative for FITC-annexin V and PI), early apoptosis (positive for FITC-annexin V), late apoptosis (double-positive for FITC- annexin V and PI), and necrosis (positive for PI) can be found in quadrants Q3,Q4,Q2 and Q1, respectively. In our study, the total percentage of |

apoptosis induction is defined as the sum of early apoptotic and late apoptotic cells.

☒ Tick this box to confirm that a figure exemplifying the gating strategy is provided in the Supplementary Information.
