# Supplementary material for: Author Correction: Targeted apoptosis of macrophages and osteoclasts in arthritic joints is effective against advanced inflammatory arthritis
Source: Nat Commun. 2026 Jul 9;17:6045. doi: 10.1038/s41467-026-75447-1 (PMC13350966; doi:10.1038/s41467-026-75447-1)
Supplement: Supplementary file 1 — Original Fig. 8, Supplementary Fig. 1 [file 41467_2026_75447_MOESM1_ESM.pdf]

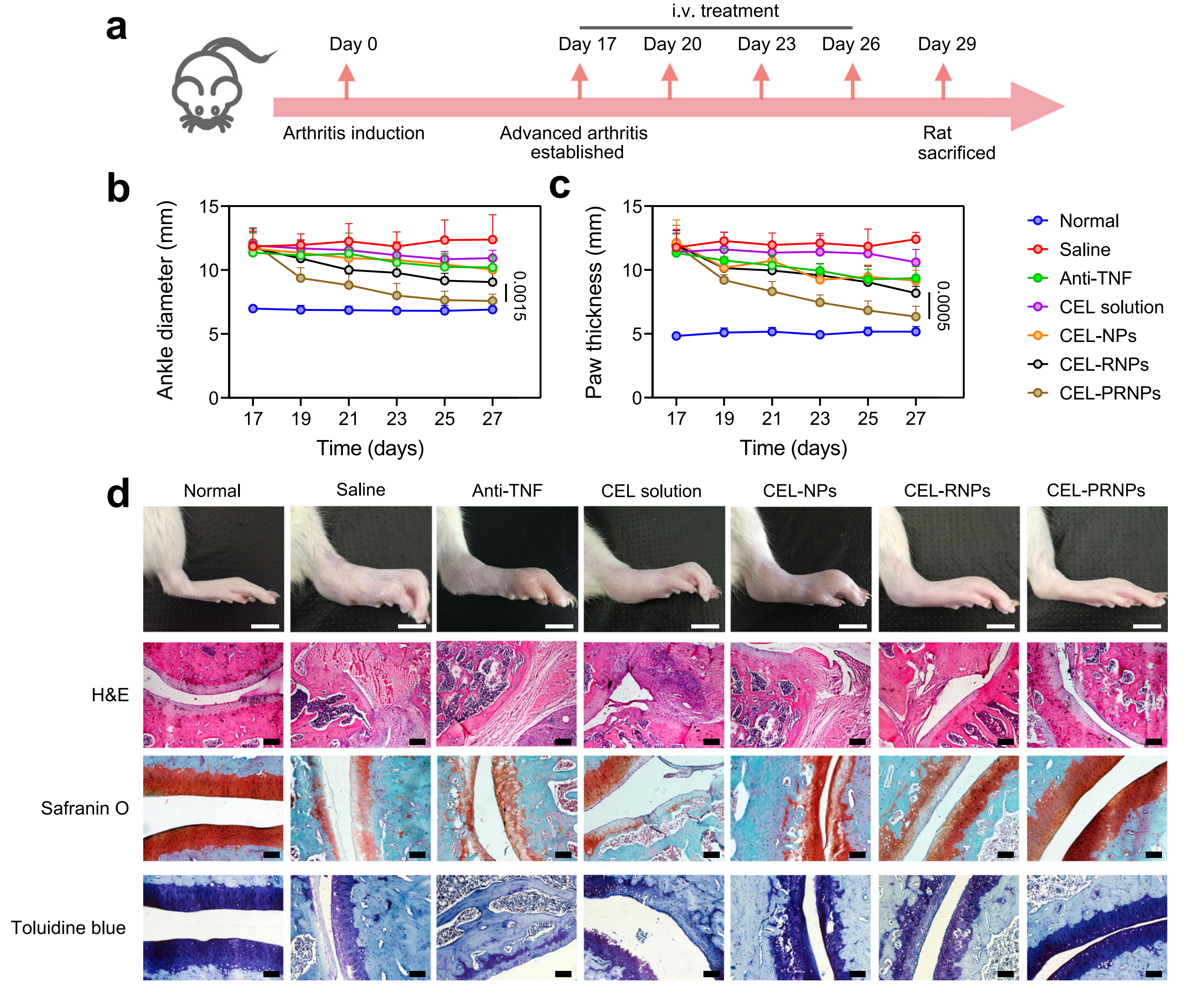

Fig. 8 Therapeutic efficacy of CEL-PRNPs in rats with advanced arthritis. a The schematic illustration of CEL-PRNPs treatment. **b**, **c** Ankle diameter (**b**) and paw thickness (**c**) of AIA rats were recorded every other day during the treatment period. Data represent mean ± SD ($n = 7$ independent animals). Statistical significance was determined by a two-sided Student's *t* test. **d** Representative photographs of hindlimbs at the endpoint of the experiment from different treatment groups (Scale bar = 10 mm); histopathology evaluation of ankle joints was identified using H&E (scale bar = 200 μm), safranin-O and toluidine blue staining (scale bar = 100 μm) ($n = 5$ independent animals). i.v. intravenous, anti-TNF anti-TNF (tumor necrosis factor) antibody, CEL celastrol, CEL-NPs CEL-loaded poly (D, L-lactide-co-glycolide) (PLGA) nanoparticles, CEL-RNPs CEL-loaded RGD peptide-modified PLGA nanoparticles, CEL-PRNPs CEL-loaded matrix metalloproteinase 9 (MMP9)-cleavable polyethylene glycol (PEG)- and RGD peptide-modified PLGA nanoparticles, H&E hematoxylin-eosin.

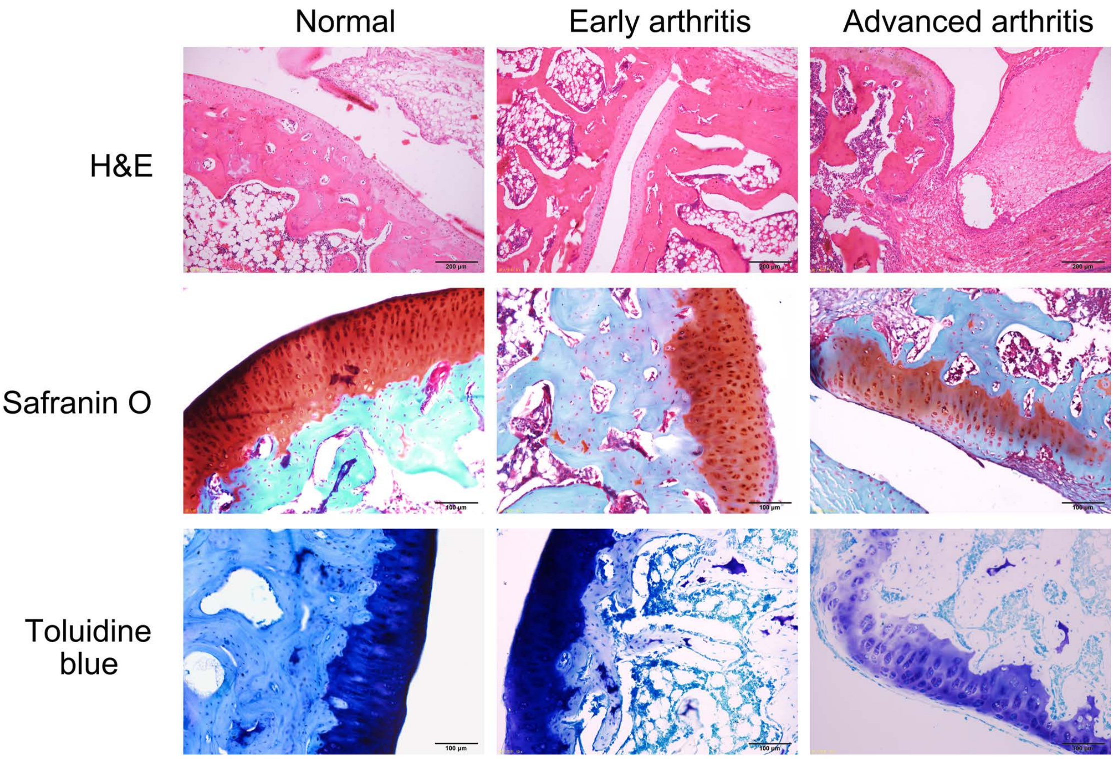

Supplementary Figure 1: Histopathology evaluation of rat ankle joints. The ankle joints from normal rats, AIA rats with early-stage arthritis and AIA rats with late-stage arthritis were stained by H&E (Scale bar = 200 μm), Safranin-O (Scale bar = 100 μm) and toluidine blue (Scale bar = 100 μm) (*n* = 5 independent animals). H&E, hematoxylin-eosin.
